# P-2080. Equipping the Front Line: HIV Education for Peer Support Counselors in Justice-Involved Communities

**DOI:** 10.1093/ofid/ofaf695.2244

**Published:** 2026-01-11

**Authors:** Lesley Simon, Dean Beals, Stan Pogroszewski, Shamile Louis, E Marq Mitchell, Jacqueline E Sherbuk

**Affiliations:** dkbmed, Brooklyn, NY; DKBmed, Brooklyn, New York; DKBmed, Brooklyn, New York; Chainless Change, Lauderhill, Florida; Chainless Change, Lauderhill, Florida; University of South Florida, Tampa, FL

## Abstract

**Background:**

Most people living in carceral facilities in the US are not receiving HIV testing, or, when indicated, pre-exposure prophylaxis. People with HIV and a history of incarceration are less likely to engage in HIV care and be virally suppressed. People incarcerated in Florida, home to the nation’s largest number of incarcerated people living with HIV, have a 7 times higher risk of acquiring or living with HIV than the general population. Counselors at Chainless Change, a peer-led re-entry agency in Broward County Florida, support a key population of ∼200 justice-involved people with HIV or at risk of acquiring HIV each year. Ending the HIV epidemic requires concentrated efforts focused on key populations.

**Table 1.**
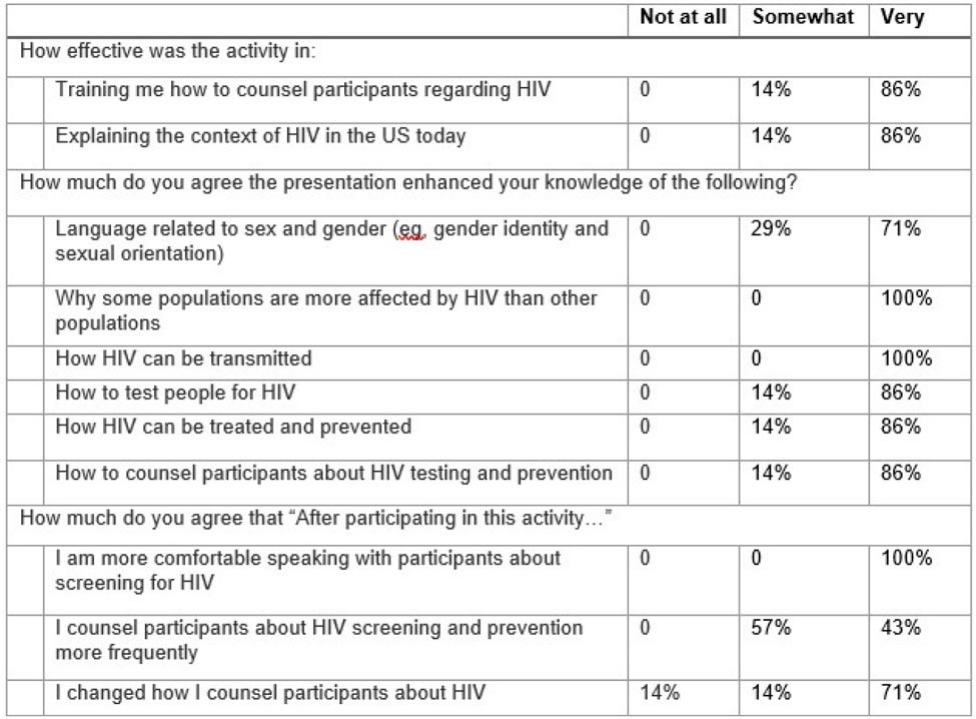
Survey responses

**Table 2.**
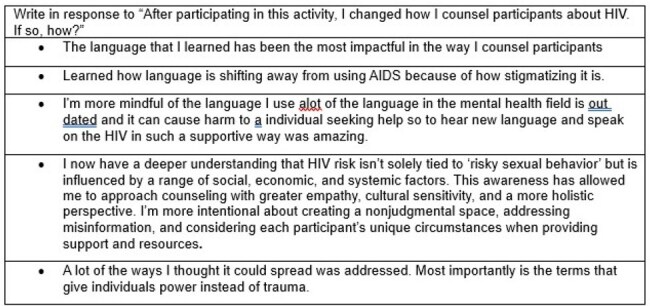
Write-in responses

**Methods:**

As part of a broad multipronged activity including an enduring accredited clinician education (n = 388 to date) and a community HIV screening event, in which 222 screenings were done, we conducted a 3-hour training session for counselors at Chainless Change that included information about HIV transmission risks, HIV prevention and treatment, and how to initiate conversations with clients about HIV testing, prevention, and treatment. We then surveyed these counselors and conducted semi-structured interviews to gain insight into the impact of the education. Qualitative data from semi-structured interviews were analyzed using thematic analysis.

**Table 3.**
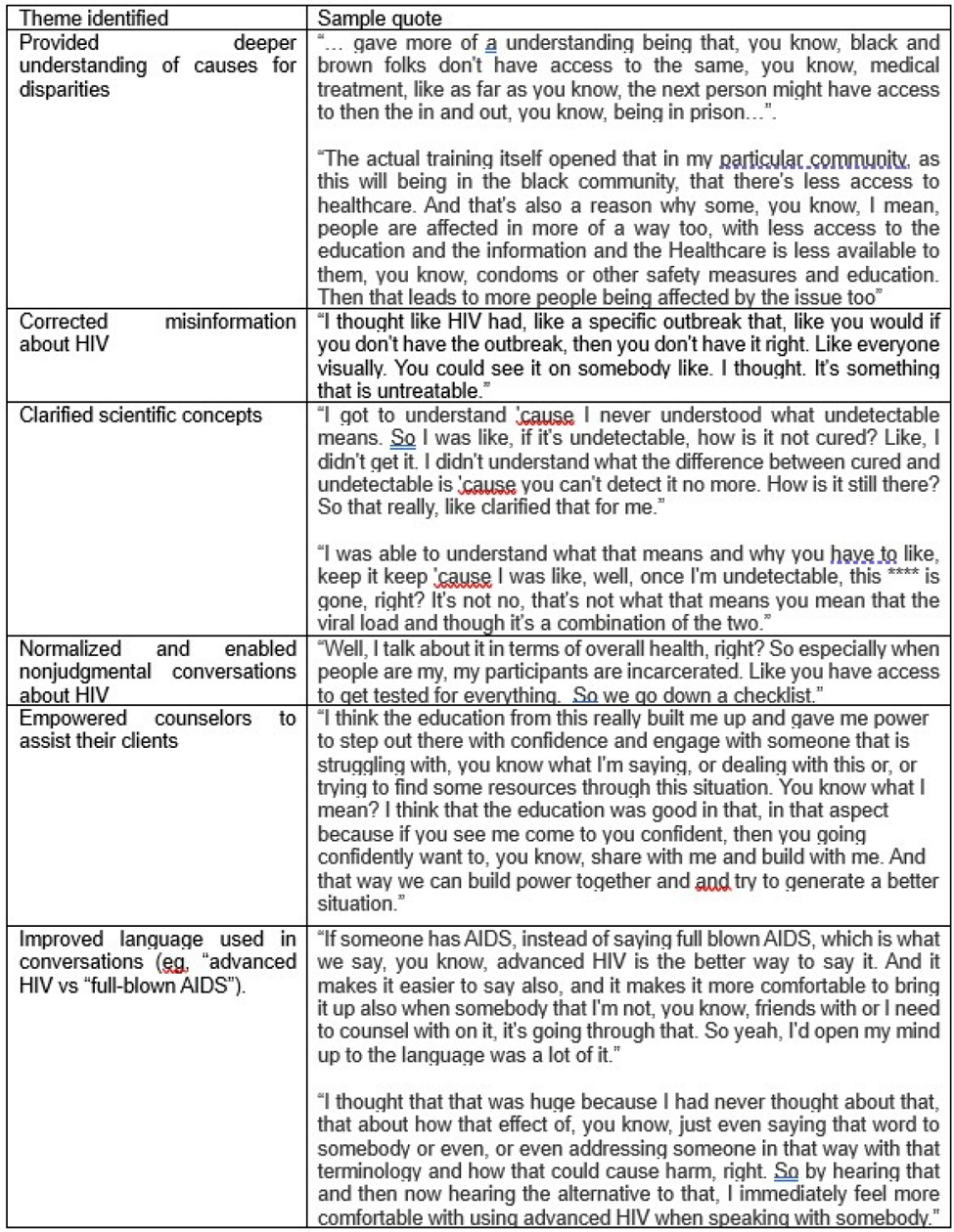
Thematic analysis.

**Figure 1.**
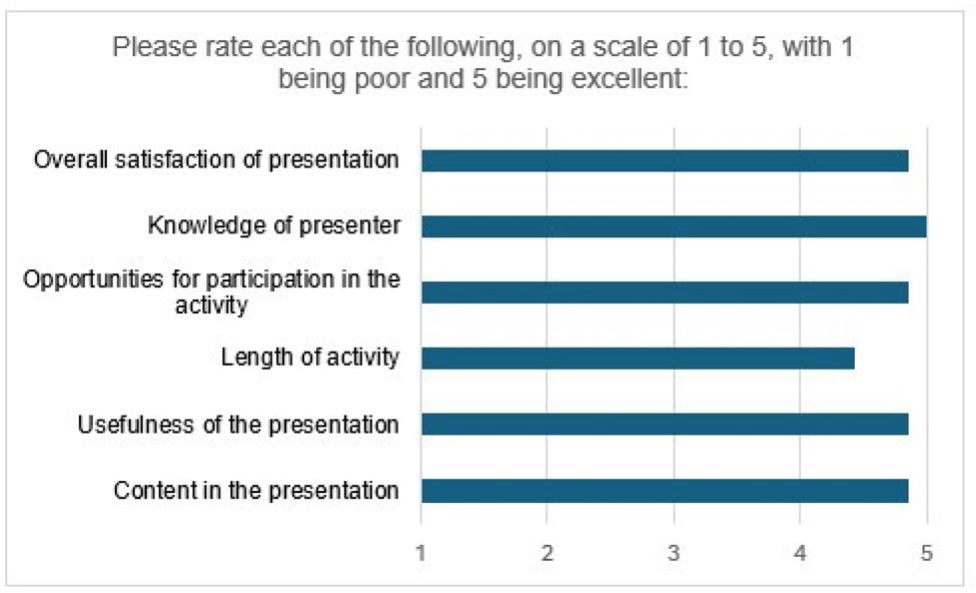
Survey responses

**Results:**

Counselors’ (n = 7) survey responses are shown in Table 1 and Figure 1 below. Counselors rated the session highly and self-reported increased knowledge of causes of HIV disparities, HIV transmission risks, HIV testing, and how to counsel their clients about HIV. All agreed that the education increased their comfort in speaking with their clients about screening for HIV. Write-in responses are shown in Table 2. Themes identified from the interviews (n = 5) are shown in Table 3.

**Conclusion:**

Education for counselors was effective in improving self-rated knowledge and was highly regarded. Counselors appreciated that the session was highly interactive and used terms and examples that were directly relatable to them, and most expressed interest in further education and requested HIV self-testing kits to provide to their clients to reduce barriers to testing.

**Disclosures:**

Lesley Simon, BA, Gilead Sciences: Independent medical education grant Dean Beals, BA, Gilead: CME Grants Stan Pogroszewski, JD, Gilead Sciences: Unrestricted CME Grant

